# *E* = *mc* ^2^: Education (*E*), medication (*m*), and conditional cash (*c* ^2^) to improve uptake of antiseizure medications in a low‐resource population: Protocol for randomized trial

**DOI:** 10.1002/epi4.12889

**Published:** 2024-01-04

**Authors:** Shawheen J. Rezaei, Fodé Abass Cissé, Mohamed Lamine Touré, Rui Duan, Dylan R. Rice, Andrew Siyoon Ham, Damien de Walque, Farrah J. Mateen

**Affiliations:** ^1^ Department of Neurology Massachusetts General Hospital Boston Massachusetts USA; ^2^ Department of Neurology Ignace Deen Teaching Hospital Conakry Guinea; ^3^ Department of Biostatistics Harvard T.H. Chan School of Public Health Boston Massachusetts USA; ^4^ Development Research Group The World Bank Washington DC USA

**Keywords:** behavioral economics, clinical trial protocol, conditional cash, epilepsy, global health, sub‐Saharan Africa

## Abstract

**Objective:**

Most people with epilepsy (PWE) could live seizure‐free if treated with one or more antiseizure medications (ASMs). The World Health Organization (WHO) estimates that 75% of PWE in low‐resource settings lack adequate antiseizure treatment. Limited education surrounding epilepsy and the out‐of‐pocket costs of ASMs in particular pose barriers to managing epilepsy in resource‐poor, low‐income settings. The aim of this study is to implement and test a novel strategy to improve outcomes across the epilepsy care cascade marked by (1) retention in epilepsy care, (2) adherence to ASMs, and (3) seizure reduction, with the measured goal of seizure freedom.

**Methods:**

A randomized, double‐blinded clinical trial will be performed, centered at the Ignace Deen Hospital in Conakry, Republic of Guinea, in Western Sub‐Saharan Africa. Two hundred people with clinically diagnosed epilepsy, ages 18 years and above, will receive education on epilepsy and then be randomized to (i) free ASMs versus (ii) conditional cash, conditioned upon return to the epilepsy clinic. Participants will be followed for 360 days with study visits every 90 days following enrollment.

**Significance:**

We design a randomized trial for PWE in Guinea, a low‐resource setting with a high proportion of untreated PWE and a nearly completely privatized healthcare system. The trial includes a conditional cash transfer intervention, which has yet to be tested as a targeted means to improve outcomes for people with a chronic neurological disorder. The trial aims to provide an evidence base for the treatment of epilepsy in such settings.

**Plain Language Summary:**

We present a clinical trial protocol for a randomized, blinded study of 200 people with epilepsy in the low‐resource African Republic of Guinea, providing an educational intervention (*E*), and then randomizing in a 1:1 allocation to either free antiseizure medication (*m*) or conditional cash (*c* ^2^) for 360 days. Measured outcomes include (1) returning to outpatient epilepsy care, (2) adherence to antiseizure medications (ASMs), and (3) reducing the number of seizures. This study is an initial look at giving small amounts of cash for desired results (or “nudges”) for improving epilepsy outcomes in the sub‐Saharan African and brain disorder contexts.

**Key points**

- Untreated epilepsy remains an important public health problem in resource‐limited settings such as the Republic of Guinea.
- There have yet to be clinical trials that evaluate the role of conditional cash in treating epilepsy.
- We provide a clinical trial protocol to test conditional cash versus free antiseizure medications (ASMs) in Guinea.
- Measured outcomes will include whether participants return to outpatient epilepsy care, adhere to ASMs, and have fewer seizures.
- This trial could provide evidence for the role of conditional cash for untreated people with epilepsy in resource‐limited settings.

## 1 INTRODUCTION

Epilepsy, the tendency to recurrent unprovoked seizures,^1^ is a chronic neurological disorder that presents across the lifespan and affects approximately 2% of the sub‐Saharan African population.^2^ Although epilepsy is usually treatable with one or more antiseizure medications (ASMs), a significant number of people with epilepsy (PWE) in low‐resource settings do not have access to or do not regularly take ASMs.^2^ Multiple organizations and investigators have noted the *epilepsy treatment gap*—defined as the difference between the number of PWE who have active epilepsy treated with ASMs versus not or the proportion of people that should be treated for epilepsy yet are not—but few studies provide an evidence base on how to change the current situation.^3^, ^4^, ^5^, ^6^ Most recently, the World Health Assembly adopted the *Intersectoral Global Action Plan on Epilepsy and Other Neurological Disorders* (“Draft Intersectoral global action plan on epilepsy and other neurological disorders 2022–2031,”^7^). This includes targets to treat more people with neurological disorders with essential medicines including ASMs for PWE.

Cascades of care are being developed to assess the delivery of care for noncommunicable diseases, similar to the approach seen in HIV/AIDS.^8^ In a cascade, specific targets are identified including: (1) ensuring >90% of PWE are aware of the diagnosis as a brain disorder, (2) retaining >90% of PWE in epilepsy care, (3) ensuring >80% of PWE take quality, appropriate chosen and dosed ASMs, and (4) ensuring seizure freedom in 70% of people treated.^9^ Of these, seizure freedom (step four) is the outcome most valued by PWE and is associated with a self‐reported quality of life equivalent to people without epilepsy. Given the high efficacy of ASMs, access to ASMs would lead to seizure freedom in a majority of PWE.

Access to ASMs remains a challenge to seizure prevention and epilepsy treatment in low‐resource settings. Epilepsy care in the Republic of Guinea, a low‐income country in Western Sub‐Saharan Africa with a 2020 gross national income of 1020 USD per capita,^10^ reflects a low‐functioning health system with few epilepsy‐trained clinicians, and high volumes of patients. Guinea has few health facilities and a nearly completely privatized healthcare system, necessitating costs to be paid “out of pocket.” PWE often go without ASMs due to multiple factors at the level of the patient, provider, and health system.

The Guinea Epilepsy Project, a prospective observational cohort study, began in 2017 by the Guinean and U.S. neurologist authors, has enrolled several 100 PWE to date.^11^, ^12^, ^13^, ^14^, ^15^ Several negative consequences of untreated and undertreated epilepsy have been observed including unintentional death; missed opportunities for school attendance of Guinean children with epilepsy^12^; poor cognitive performance; frequent unintentional injuries; epilepsy stigma^16^; high rates of traditional healing injuries including cuttings, instrumentation, and animal sacrifices^11^; and large losses of personal wealth on ineffective treatment options.^17^ Similar to other low‐resource locations, a high number of PWE do not take ASMs and have suffered serious consequences as a result, including death from unintentional injury or status epilepticus.^13^

As a response to this tragic situation, the study team proposes to compare two ways to deliver epilepsy care to people with epilepsy (PWE) in a low‐income setting. Although several lines of evidence suggest that both these interventions will lead to improved outcomes,^18^, ^19^, ^20^ they have not been directly tested in low‐resource populations with low literacy. *Conditional cash transfer* (CCT) interventions in particular have not been systematically tested in PWE or in people with any chronic neurological disorder. CCT interventions grant individuals a sum of money on the condition that they fulfill a predetermined task. In this way, CCT interventions create a “nudge,” or an economic incentive, for taking a desired course of action. These interventions differ notably from *unconditional cash transfer* (UCT) interventions, which simply provide direct payment to individuals without any behavioral nudges. As such, CCT interventions uniquely serve as an important lever for both alleviating the financial burden of poverty and incentivizing people to act for the benefit of their own health.

A global scoping review of randomized trials with health‐focused CCT interventions found most CCT interventions benefit health outcomes and have the potential to change patient behaviors for a range of disease areas.^21^ However, CCT interventions are yet to be tested for neurological conditions. Moreover, CCT has limited testing for health outcomes in West African countries, such as Guinea, where traditional healing, high stigma, poverty, and misbeliefs about the origins of epilepsy prevail.^22^, ^23^, ^24^, ^25^ CCT interventions could therefore enable epilepsy patients to break out of a cycle of poverty that can accompany high seizure burdens and resultantly, an inability to attend school or work.

### 1.1 Trial design

**FIGURE 1:**
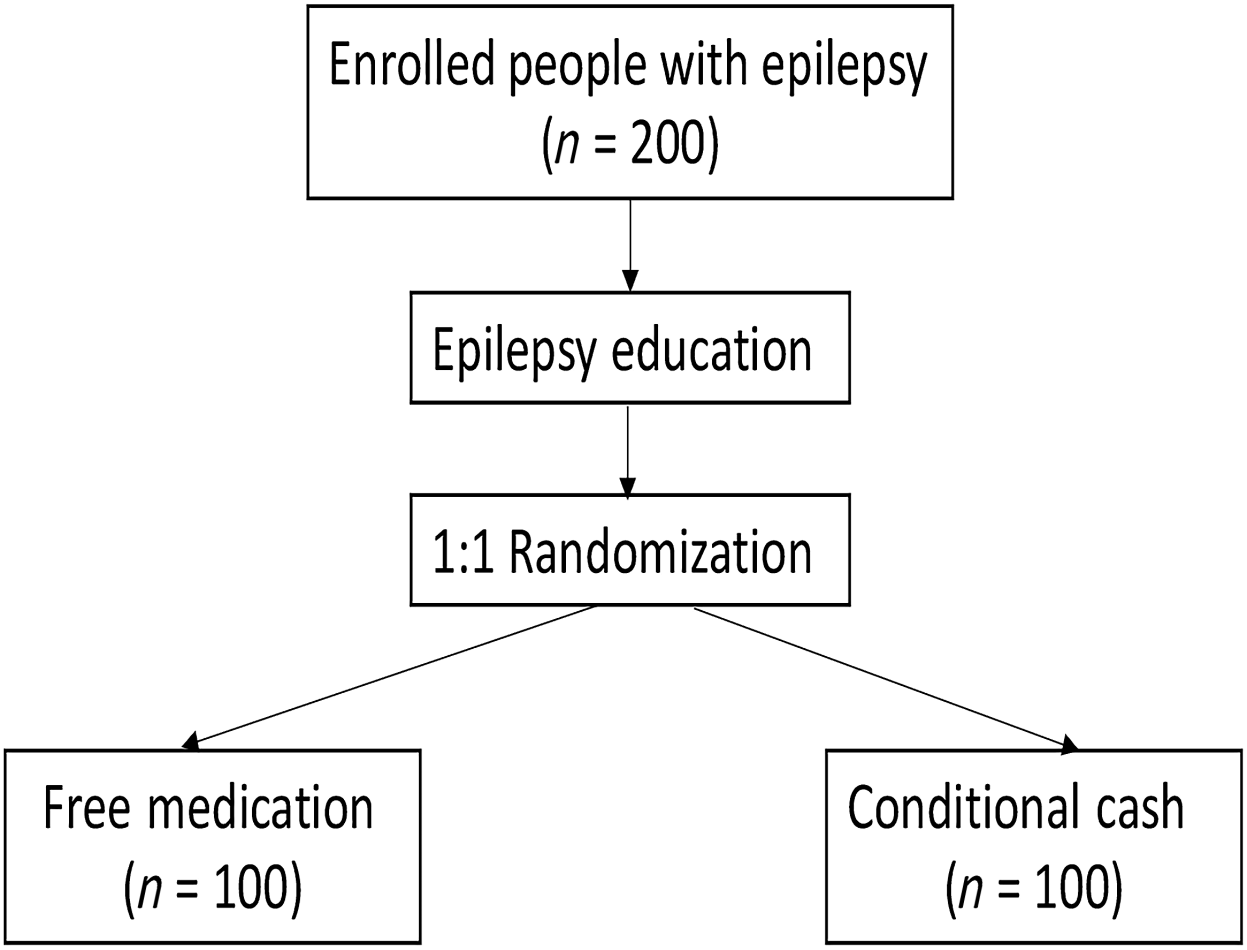
Study enrollment and intervention randomization flow diagram.

A randomized, double‐blinded clinical trial will be performed, including multiple healthcare facility sites in rural and urban Guinea but headquartered at the Ignace Deen Hospital in the capital city, Conakry. Two hundred PWE, clinically diagnosed by a Guinean and a U.S. neurologist, ages 18 years and above, will be enrolled and randomized 1:1 to (i) education and medication, or (ii) education and CCT (Figure 1). All participants will receive epilepsy education. Participants will be interviewed and examined by the Guinean–U.S. study team of neurologists at each visit. Follow‐up visits every 90 days until day 360 (approximately 1 calendar year) will assess the intervention on participants' clinical and socioeconomic outcomes. The recurring 90‐day follow‐up visits are considered beneficial to participants who may not access epilepsy care in any other way while also providing a sufficiently long timeline to study the sustainability of this intervention.

### 1.2 Objectives

The primary objective is to implement and test a novel strategy to improve outcomes across the epilepsy care cascade marked by (1) retention in epilepsy care, (2) adherence to ASMs, and (3) seizure reduction with the measured goal of seizure freedom.

The first aim is to compare free ASMs vs. conditional cash using the epilepsy cascade of care. We hypothesize that the provision of free medication versus conditional cash will lead to (1) similar retention in epilepsy care—90% of PWE—at 360 days (Hypothesis 1a), (2) 80% of PWE regularly taking ASMs at 360 days (Hypothesis 1b), and (3) 65% of PWE achieving seizure freedom 360 days postenrollment (Hypothesis 1c).

The secondary aim is to detail facilitators and barriers to free medication versus conditional cash to improve seizure freedom among PWE. We hypothesize that barriers and facilitators to the uptake of each intervention strategy can be learned through careful, structured documentation by healthcare workers during study visits using an investigator‐designed written intake form as well as free‐text responses, serving as foundational information for a future larger‐scale implementation study in low‐resource populations.

## 2 METHODS: PARTICIPANTS, INTERVENTIONS, AND OUTCOMES

Institutional Review Board approval will be obtained through the Ignace Deen Hospital in Guinea and the U.S. study team's institution.

### 2.1 Study setting

The Ignace Deen Hospital is one of two teaching hospitals in Conakry, receiving several thousand cases of epilepsy per year. Additional recruitment and enrollment are planned to occur in Kankan, Forecariah, and Dubreka regional health facilities. There are five practicing neurologists in Guinea. Table 1 displays summary statistics from a prior cohort study we conducted with 297 Guinean PWE whose characteristics are expected to resemble future trial participants.

**TABLE 1:** Epilepsy patients, Republic of Guinea (*n* = 297).

	Female (*n* = 159)	Male (*n* = 138)	Children (<18 years, *n* = 148)	Adults (> = 18 years, *n* = 149)	Full cohort (*n* = 297)
Mean age (years; range)	20.4 (1–66)	18.6 (0.8–62)	8.5 (0–17)	30.5 (18–66)	19.6 (0.8–66)
Highest education (*n*)					
No school	35	32	48	19	67
Primary school	52	50	72	30	102
Middle school	23	21	18	26	44
High school	17	17	5	29	34
University	30	15	1	44	45
Was water unavailable for at least 1 day in the past 2 weeks? (% yes)	47	37	40	44	43
Has previously taken ASMs (% yes)	89	78	84	84	84
Currently taking ASMs (% yes)	70	54	60	65	63
Annual mean household income per capita (USD)	639	924	603	975	770
Duration of epilepsy, years (mean)	7.6	7.2	3.5	11.1	7.4
Seizure frequency in past month (mean)	3.0	4.1	4.1	2.8	3.5
Ever injured due to seizures (% yes)	58	51	35	74	55
Ever used non‐allopathic treatments (*n*)					
Traditional medicine	92	70	78	84	162
Prayer	29	26	29	26	55
Special diets	16	3	4	15	19
ASMs ever taken (*n*)					
Carbamazepine	70	51	44	77	121
Benzodiazepine	6	7	6	7	13
Levetiracetam	28	19	29	18	47
Phenobarbital	60	33	41	52	93
Phenytoin	0	1	0	1	1
Sodium valproate	58	35	58	35	93
Other ASM	10	11	5	16	21
Previous brain imaging (% yes)					
CT	42	33	37	38	38
EEG	49	49	55	42	49
MRI	5	2	4	3	4

*Note*: Gray shades denote the title of the columns.

### 2.2 Eligibility criteria

PWE will be screened in person and enrolled on a “first come, first served” basis. The goal is to enroll a person with active epilepsy. The person will have therefore experienced two or more recurrent, unprovoked seizures in the past year (inclusion criterion) and not have had any other diagnosis that can better explain the symptoms, as evaluated by the study neurologists (one Guinean and one U.S.). Specifically, patients with exclusively febrile seizures, pseudo‐seizures, and other movement disorders or episodic events that are not primary epilepsy are not eligible. The study will include a brain MRI such that secondary seizures from a space‐occupying lesion (secondary to tumor, stroke) can be detected and referred to usual care but not enrolled. Similarly, seizures that are provoked (e.g., alcohol withdrawal) will be queried through the intake forms and corroborated by physical examination and extant medical records.

Inclusion criteria are (1) resident of Guinea, 18 years or older, presenting to one of the study sites in Guinea; (2) able to provide informed consent or have a next of kin proxy provide informed consent when appropriate; (3) experienced two or more recurrent, unprovoked seizures in the past year; (4) able to provide a clinical history or have a next of kin provide it; (5) not taking a daily ASM for epilepsy for the past 30 days or more for any reason; (6) willing to have his/her data sent anonymously to the U.S. study site; and (7) anticipated to be able to return for follow‐up visits for 360 days.

Exclusion criteria are (1) any other diagnosis that can better explain the symptoms, making the diagnosis of primary, active epilepsy unlikely; (2) unable to undergo the study procedures; (3) age less than 18 years old due to the need to measure several self‐reported outcomes; (4) unable to provide informed consent (or have an appropriate proxy informed consent); (5) currently pregnant or lactating; (6) secondary seizures, for example, due to a condition that requires imminent treatment (e.g., malignancy, alcohol withdrawal); (7) critically ill; (8) exclusively pseudo‐seizures/nonepileptic behavioral events; (9) unwilling to take an ASMs for epilepsy; (10) already treated with ASMs for other purposes (e.g., mood, pain); (11) known to be unable to return for follow‐up visits.

The investigators will monitor participants' health throughout the study and re‐evaluate eligibility. For example, a woman may become pregnant or a participant may have a new illness (e.g., malaria). It will be ensured that all pregnant women have access to clinical care and an ASM (such as levetiracetam or lamotrigine) that is associated with the lowest fetal risk (whether she remains in the study or is withdrawn). Changes in health status and medical risk will be recorded, and the study team will adjudicate the event for administrative withdrawal of the participant. All withdrawn participants will have access to usual care following study discontinuation.

### 2.3 Informed consent

Screening will be performed by Guinean investigators with mentorship and collaboration with the U.S. team (Appendix S1) and will include a brain MRI to rule out other diagnoses (e.g., tumors, untreated significant hydrocephalus). An estimated 50% of the enrollment will occur in Conakry and 50% at rural and peri‐urban sites.

## 3 INTERVENTIONS

### 3.1 Explanation for the choice of comparators

Participants randomized to the CCT arm will still be given an ASM recommendation and advice on purchasing ASMs consistent with the best available neurological care. The decision to create two arms will enable the study team to assess the effectiveness of each intervention without using a noninterventional control group. Using a control group comparator without any intervention was considered unethical. All participants will have access to local diagnostic testing as needed.

### 3.2 Intervention description

The education intervention will be given to each participant upon study enrollment in the form of a prerecorded, standardized video, made by the investigators, lasting approximately 30 min. The video is designed for people with low literacy and will answer the following basic questions: [What is a seizure? What is epilepsy? Why does epilepsy occur? Why is epilepsy a brain disorder? What works to treat epilepsy? What does not work to treat epilepsy? Why are ASMs important?] The video will be available in local languages (Sousou and Malinké), English, and French and shown on electronic tablets. A question‐and‐answer period, led by the study neurologists, will follow with no limitations on the amount of time. A flyer providing basic, written information on the education intervention will be given.

ASMs will be provided free of charge and prescribed for the participants in the study arm that includes the medication intervention. The ASM choice will be determined at the discretion of the neurologist in the context of the patient's history. There is no one best ASM for all patients, and most ASMs provide similar efficacy but dissimilar side effects and safety. There will be a total of seven possible ASMs to be used.

CCT will be conditioned on the return for follow‐up visits to the outpatient epilepsy clinic for scheduled appointments (±7 days every 90 days). Each visit for those in the CCT groups will entail the provision of 15 USD (given in the equivalent of Guinean Francs) to participants. This amount is anticipated to be a “nudge” (i.e., mildly incentivizing) as it exceeds the general cost of transportation for a return visit in the area (~5 USD on average) and ASM (which approximately costs ~4–10 USD for a monthly dosage in Guinea).^11^ It is important to note that this nudge will ensure participants in the CCT arm can afford the ASM, which enables study staff to assess outcomes from behavioral change rather than affordability considerations. The transfer will also be conditional on presence at visits, not on seizure outcomes. This ensures that participants are not penalized for aspects of their illness that are beyond their control.

### 3.3 Outcomes

The primary outcome of the study is adherence to neurology clinic appointments. This is defined as the number of appointments attended as scheduled and on time by the participant, as reported by the investigator.

Secondary outcomes are as follows:

1. Percent of PWE who achieve seizure freedom.
2. Seizure frequency reduction: The percentage decrease in number of monthly seizures from baseline to the study endpoint of 360 days.

A complete list of outcomes can be found in Table 2.

**TABLE 2:** Primary, secondary, and exploratory outcomes of interest.

Outcome category	Outcome	Definition	Method of measuring
Primary	Adherence to Neurology Clinic Appointments	The number of appointments attended as scheduled and on time by the participant	Investigator reported
Secondary	Seizure freedom	% of PWE who achieve seizure freedom, or the ability to live without seizures of any type	Seizure diary counts
Secondary	Seizure frequency reduction	The percentage decrease in the number of monthly seizures from baseline to the study endpoint of 360 days	Seizure diary counts
Exploratory	Quality of life in epilepsy	A standardized scale and subjective report of physical, social, and psychological well‐being by a person with epilepsy	Patient/parent reported
Exploratory	Number of visits to traditional healers	Self‐reported number of visits to non‐allopathic providers for epilepsy management	Patient reported
Exploratory	Self‐reported adherence to ASMs	The number of pills left in the medication bottle, determined as a medication possession ratio	Investigator Counted
Exploratory	Stigma scale in epilepsy	A standardized scale and subjective report of stigma perceived by a person with epilepsy	Patient reported
Exploratory	Days attended at work or school	Self‐reported days attending school or work by a person with epilepsy of the past 90 days	Patient reported
Exploratory	Number of unintentional injuries due to seizures	Including burns, fractures, head injury, and related injuries in the past 90 days	Patient reported
Exploratory	Number of status epilepticus events	Reported by participant and family member in a log, articulating a seizure episode lasting >5 min	Patient reported

### 3.4 Participant timeline

The timeline of screening, enrolment, and interventions is provided in Table 3. The interventions will be provided at follow‐up visits taking place every 90 days. The baseline visit will include a neurologist's description of seizure history, including seizure semiology, epilepsy diagnosis, and etiology of epilepsy to the degree possible given resource limitations. Structured forms will be used. Several measures will be collected during follow‐up visits, including detailed seizure information, quality of life surveying, medication possession, stigma scale surveying using the Stigma Scale of Epilepsy (SSE),^26^ health visit history, school/work attendance, and injury surveying.

**TABLE 3:** Schedule of tests and events at each study visit.

	Screening	Baseline	90	180	270	360
Study visit		1	2	3	4	5
Informed consent	×					
Assess inclusion and exclusion criteria	×	×				
Medical history	×	×				×
Seizure diary		×	×	×	×	×
Concomitant medication history		×		×		×
Quality of Life in Epilepsy‐31 scale		×	×	×	×	×
Consumption and household assets module		×				×
Stigma Scale of Epilepsy		×	×	×	×	×
Medication possession ratio, as appropriate based on assigned arm			×	×	×	×
Health visit history (allopathic and traditional)		×	×	×	×	×
School/work attendance log		×	×	×	×	×
Injury survey		×	×	×	×	×
Status epilepticus query		×	×	×	×	×
Body weight, height, heart rate		×				×
Urine pregnancy test (for people of childbearing potential), *as needed	×	*	*	*	*	*
Clinical labs (including serum Na+, glucose), *as needed	*	×	*	*	*	*
Brain MRI	×					×
Conditional cash allocation, as appropriate based on assigned arm		×	×	×	×	×
Vitality status assessment			×	×	×	×

### 3.5 Recruitment

Participants will be recruited via (1) clinicians' referrals, (2) referrals through in‐person meetings with traditional healers, and (3) if needed, advertisements through *Radio Television Guinea* and local Conakry‐based radio and television stations that reach most of the Guinean population. Traditional healers will be leveraged for recruitment purposes but will not serve as investigators or otherwise monitor study interventions. Most participants will be recruited by Guinean neurologists, as in preliminary studies.

## 4 ASSIGNMENT OF INTERVENTIONS: BLINDING

### 4.1 Who will be blinded?

Participants will be blinded to the primary study hypothesis. The Guinean site collaborators providing the intervention will be blinded to the primary study hypothesis.

## 5 DATA COLLECTION AND MANAGEMENT

### 5.1 Plans for assessment, collection of outcomes, and data management

Data will be collected from the baseline visit and follow‐up visits (Table 3). An enrollment survey is provided in Appendix S2. All investigators will receive research ethics and data quality control training to ensure adherence to proper documentation and data collection. Data quality control measures will be implemented by the U.S. and Guinean staff on a weekly basis to ensure accuracy. Missing data will be addressed throughout the study. Use of REDCap™ with forced responses to progress to the next page will be used for surveys to avoid missingness. Other data points that are missing in a systematic way will be re‐evaluated for any clinical or cultural reasons. No data imputation will occur.

### 5.2 Plans to promote participant retention and complete follow‐up

The study investigators will provide a careful explanation of the importance of follow‐up visits before a prospective participant gives informed consent. Participants will be asked to follow up in person and reminded of upcoming visits approximately a week in advance. Participants will also be given access to a study phone number and allowed to attend their visit ±7 days of the scheduled date to accommodate personal, work, and/or public transit schedules and further promote retention.

## 6 STATISTICAL METHODS

We choose a randomized two‐arm design with an equal sample size in the arm with the provision of conditional cash and the arm with the provision of free medication. Our sample size calculation is based on Hypothesis 1a.

To investigate Hypothesis 1a, we will first test the null hypothesis that the retention rate of the group with the provision of conditional cash is less than or equal to 90% using a non‐inferiority one‐sample proportion test. We will then test the null hypothesis that the provision of conditional cash will lead to a retention rate no less than the retention rate of free medication (controlling for medication type) using a noninferiority two‐sample proportion test. Since there are two hypotheses, in the sample calculation, we applied the Bonferroni correction to ensure a familywise error rate of 0.05.^27^ Given the existing studies and preliminary results, we expect that the retention rate of the group with the provision of conditional cash will have a retention rate higher than 90%. This reflects financial incentive clinical trials in other diseases where the intervention improves retention in care, particularly studied in HIV/AIDS.^28^, ^29^

Assuming it is 90%, to ensure a statistical power of 80%, with a noninferiority margin of 8.5%, the sample size of the arm with the conditional cash required is 98.^30^ We will then test the null hypothesis that the provision of conditional cash will lead to a retention rate no less than the retention rate of free ASMs using a noninferior two‐sample proportion test. Assuming the conditional cash group has 90% of PWE who will return at 12 months, with a noninferiority margin of 12%, to ensure statistical power of 80%, the sample size of the arm with the conditional cash required is 99.

For Hypothesis 1b, we will use a one‐sample proportion test to test the null hypothesis that the percentage of PWE regularly taking ASMs at 12 months in the conditional cash arm is less than or equal to 85%, and we will use a two‐sample proportion test for the null hypothesis that the percentage of PWE regularly taking ASMs in the conditional cash arm is less than or equal to the percentage of PWE regularly taking ASMs in the free medication arm. For Hypothesis 1c, we will use a one‐sample proportion test to test the null hypothesis that the percentage of PWE with seizure freedom at 12 months in the conditional cash arm is less than or equal to 65%, and we will use a two‐sample proportion test for the null hypothesis that the percentage of seizure freedom in the conditional cash arm is less than or equal to the percentage of seizure freedom in the free medication arm.

To leverage the longitudinal observations collected during follow‐up visits at 90, 180, 270, and 360 days, we will use a generalized linear mixed effects model which allows us to study the association between multiple baseline variables (e.g., including the intervention options, education level, medication status, ASM type, distance to the study clinics) while accounting for the correlation between longitudinal observations.

## 7 OVERSIGHT AND MONITORING

### 7.1 Data monitoring committee

The study will convene a Data Safety and Monitoring Board (DSMB) quarterly throughout the trial period. The DSMB will be composed of Guinean, U.S., and other global experts, including neurologists and biostatisticians, to review adverse events, trial progress, and data on the outcomes of interest. Adverse effects of the ASMs will be recorded. The DSMB may vote to unblind and withdraw a participant in the event of a serious adverse effect. Participants will be advised of any abnormal head imaging results performed by the study and referred for appropriate clinical care.

### 7.2 Dissemination plans

Transparency on processes and protocols and dissemination of trial data will be enabled through sharing of all survey instruments, data collection materials, data analysis plans, and de‐identified data. These will be made available in academic peer‐reviewed journals and institutional research websites with final data available free online.

## 8 DISCUSSION

Our study provides a concrete plan to disaggregate issues contributing to the epilepsy treatment gap and target simple interventions addressing three major presumed reasons for the treatment gap: low epilepsy awareness, limited access to ASMs, and absolute poverty. Our approach provides a new pathway and model for clinicians and researchers to consider this problem. This study comes at a time when the international community is searching for effective mechanisms of alleviating the burden of epilepsy globally. The *Intersectoral Global Action Plan on Epilepsy and Other Neurological Disorders 2022–2031* emphasizes the need for concerted, interdisciplinary action to enhance epilepsy care and treatment in the near term (“Draft Intersectoral global action plan on epilepsy and other neurological disorders 2022–2031,”^7^). CCT interventions, if proven to be effective for epilepsy care, could serve as a powerful tool for these global efforts.

There are several strengths to this proposed study. The study design is both ethical and appropriate for low‐resource populations and, if successful, could be programmatically scaled to other locations. We also propose the first test of CCT, a method that has been used in other conditions, such as HIV/AIDS^31^ and TB,^32^ and in vaccination campaigns, to address low uptake. The use of CCT is controversial and untested in PWE but could serve as a poverty‐reduction intervention in addition to improving health outcomes among PWE. Though commonly tested for other health‐focused outcomes and in other regions, there is no published research on CCT programs in neurological disorders to our knowledge and only a limited sample of tested CCT interventions in West Africa.^33^, ^34^

There are also several limiting factors to the study design. Though participants are blinded to the hypothesis of the study, there is a possibility of participants disclosing information on their allocation to other participants, which would reduce allocation blindness. There is also a risk of differential attrition between the study arms. Several study outcomes are participant‐reported, which require clear and proactive communication with participants.

In general, large medication trials for neurological disorders on the African continent remain surprisingly rare. Testing interventions that can improve and save lives is overdue in the field of neurology and, even more so, in resource‐limited settings. This randomized trial, enrolling an anticipated 200 PWE in West Africa, will establish an evidence base for a leading problem in neurological care in low‐resource settings. The trial is designed for appropriateness in Guinean PWE with health equity and local standards of care considered. Inherent to the study is capacity building for epilepsy treatment and management among patients, families, and clinicians who will be actively involved in the *E* = *mc* ^2^ protocol. The motivation of the trial design is to improve the overall situation of epilepsy care in Guinea, that is, to do no harm and leave no patient behind. As such, all trial participants are anticipated to benefit from their allocated study intervention, even if the study hypotheses are not supported.

## AUTHOR CONTRIBUTIONS

Study idea: FJM. Data collection and management: FAC, MLT. Data analysis: SJR, RD, DRR. Manuscript writing: SJR, FJM. Manuscript edits, revisions, and final approval: SJR, FAC, MLT, RD, DRR, DdW, and FJM.

## CONFLICT OF INTEREST STATEMENT

Dr. Damien de Walque: The findings, interpretations, and conclusions expressed in this paper are entirely those of the authors. They do not necessarily represent the views of the World Bank, its Executive Directors, or the countries they represent. Dr. Mateen has received research funding from EMD Serono, Genentech, Horizon Therapeutics, and Novartis, unrelated to this work. All other authors report no disclosures or conflicts of interest.

## ETHICAL APPOVAL

We confirm that we have read the Journal's position on issues involved in ethical publication and affirm that this report is consistent with those guidelines.

## Supporting information


Appendix S1.

Appendix S2.

## Data Availability

Data sharing is not applicable to this article as no new data were created or analyzed in this study.

## References

[1] Rao VR , Lowenstein DH . Epilepsy. Curr Biol. 2015;25:R742–R746. 10.1016/j.cub.2015.07.072 26325130

[2] Newton CR , Garcia HH . Epilepsy in poor regions of the world. Lancet Lond Engl. 2012;380:1193–1201. 10.1016/S0140-6736(12)61381-6 23021288

[3] Meyer A‐C , Dua T , Ma J , Saxena S , Birbeck G . Global disparities in the epilepsy treatment gap: a systematic review. Bull World Health Organ. 2010;88:260–266. 10.2471/BLT.09.064147 20431789 PMC2855595

[4] Mogal Z , Aziz H . Epilepsy treatment gap and stigma reduction in Pakistan: a tested public awareness model. Epilepsy Behav. 2020;102:106637. 10.1016/j.yebeh.2019.106637 31805506

[5] Owolabi LF , Shehu MY , Shehu MN , Fadare J . Pattern of neurological admissions in the tropics: experience at Kano, Northwestern Nigeria. Ann Indian Acad Neurol. 2010;13:167–170. 10.4103/0972-2327.70875 21085525 PMC2981752

[6] Wagner RG , Kabudula CW , Forsgren L , Ibinda F , Lindholm L , Kahn K , et al. Epilepsy care cascade, treatment gap and its determinants in rural South Africa. Seizure. 2020;80:175–180. 10.1016/j.seizure.2020.06.013 32593141 PMC7443697

[7] WHO . Draft Intersectoral global action plan on epilepsy and other neurological disorders 2022–2031. [WWW Document] 2022 Accessed October 19, 2022. https://www.who.int/news/item/28‐04‐2022‐draft‐intersectoral‐global‐action‐plan‐on‐epilepsy‐and‐other‐neurological‐disorders‐2022‐2031.

[8] Kay ES , Batey DS , Mugavero MJ . The HIV treatment cascade and care continuum: updates, goals, and recommendations for the future. AIDS Res Ther. 2016;13:35. 10.1186/s12981-016-0120-0 27826353 PMC5100316

[9] Mateen FJ . A cascade of care for people with epilepsy: learning from "HIV/AIDS 90‐90‐90". Gates Open Res. 2019;3:1502. 10.12688/gatesopenres.13043.2 31508582 PMC6712888

[10] World Bank . GNI per capita, Atlas method (current US$). 2023 [WWW Document] Accessed September 24, 2023. https://data.worldbank.org/indicator/NY.GNP.PCAP.CD

[11] Anand P , Othon GC , Sakadi F , Tassiou NR , Hamani ABD , Bah AK , et al. Epilepsy and traditional healers in the Republic of Guinea: a mixed methods study. Epilepsy Behav. 2019;92:276–282. 10.1016/j.yebeh.2019.01.017 30731293 PMC6433505

[12] Fitts W , Rahamatou NT , Abass CF , Vogel AC , Ghislain AH , Sakadi F , et al. School status and its associations among children with epilepsy in the Republic of Guinea. Epilepsy Behav. 2019;97:275–281. 10.1016/j.yebeh.2019.05.040 31260925 PMC6702082

[13] Jang M , Sakadi F , Tassiou NR , Abass CF , Grundy SJ , Woga A , et al. Impact of poorly controlled epilepsy in the Republic of Guinea. Seizure. 2018;61:71–77. 10.1016/j.seizure.2018.07.018 30114675 PMC6168342

[14] Sokolov E , Abdoul Bachir DH , Sakadi F , Williams J , Vogel AC , Schaekermann M , et al. Tablet‐based electroencephalography diagnostics for patients with epilepsy in the west African Republic of Guinea. Eur J Neurol. 2020;27:1570–1577. 10.1111/ene.14291 32359218 PMC8830803

[15] Williams JA , Cisse FA , Schaekermann M , Sakadi F , Tassiou NR , Hotan GC , et al. Smartphone EEG and remote online interpretation for children with epilepsy in the Republic of Guinea: quality, characteristics, and practice implications. Seizure. 2019;71:93–99. 10.1016/j.seizure.2019.05.025 31229939 PMC6783351

[16] Rice DR , Cisse FA , Djibo Hamani AB , Tassiou NR , Sakadi F , Bah AK , et al. Epilepsy stigma in the Republic of Guinea and its socioeconomic and clinical associations: a cross‐sectional analysis. Epilepsy Res. 2021;177:106770. 10.1016/j.eplepsyres.2021.106770 34619642 PMC8557132

[17] Rice DR , Sakadi F , Tassiou NR , Vogel AC , Djibo Hamani AB , Bah AK , et al. Socioeconomic associations of poorly controlled epilepsy in the Republic of Guinea: cross‐sectional study. Trop Med Int Health. 2020;25:813–823. 10.1111/tmi.13407 32324940

[18] Mbuba CK , Ngugi AK , Newton CR , Carter JA . The epilepsy treatment gap in developing countries: a systematic review of the magnitude, causes, and intervention strategies. Epilepsia. 2008;49:1491–1503. 10.1111/j.1528-1167.2008.01693.x 18557778 PMC3573323

[19] Ranganathan M , Lagarde M . Promoting healthy behaviours and improving health outcomes in low and middle income countries: a review of the impact of conditional cash transfer programmes. Prev Med. 2012;55(Suppl):S95–S105. 10.1016/j.ypmed.2011.11.015 22178043

[20] Sander JW . The use of antiepileptic drugs—principles and practice. Epilepsia. 2004;45(Suppl 6):28–34. 10.1111/j.0013-9580.2004.455005.x 15315513

[21] Rezaei SJ , de Walque D , Mateen FJ . Conditional cash transfers to improve health‐focused outcomes: a global scoping review. Glob Public Health. 2022;1–18:3368–3385. 10.1080/17441692.2022.2092186 35727705

[22] Gugssa SA , Haidar J . Knowledge, attitude, and practice towards epilepsy among religious cleric and traditional healers of Addis Ababa, Ethiopia. Seizure. 2020;78:57–62. 10.1016/j.seizure.2020.03.006 32203881

[23] Keikelame MJ , Swartz L . “A thing full of stories”: traditional healers' explanations of epilepsy and perspectives on collaboration with biomedical health care in Cape Town. Transcult Psychiatry. 2015;52:659–680. 10.1177/1363461515571626 25680366 PMC4552613

[24] Maiga Y , Albakaye M , Diallo LL , Traoré B , Cissoko Y , Hassane S , et al. Current beliefs and attitudes regarding epilepsy in Mali. Epilepsy Behav. 2014;33:115–121. 10.1016/j.yebeh.2014.02.031 24657502

[25] Millogo A , Ngowi AH , Carabin H , Ganaba R , Da A , Preux P‐M . Knowledge, attitudes, and practices related to epilepsy in rural Burkina Faso. Epilepsy Behav. 2019;95:70–74. 10.1016/j.yebeh.2019.03.006 31026786 PMC6686174

[26] Fernandes PT , Salgado PCB , Noronha ALA , Sander JW , Li LM . Stigma scale of epilepsy: validation process. Arq Neuropsiquiatr. 2007;65(Suppl 1):35–42. 10.1590/s0004-282x2007001000006 17581666

[27] Bland JM , Altman DG . Multiple significance tests: the Bonferroni method. BMJ. 1995;310:170. 10.1136/bmj.310.6973.170 7833759 PMC2548561

[28] Fahey CA , Njau PF , Katabaro E , Mfaume RS , Ulenga N , Mwenda N , et al. Financial incentives to promote retention in care and viral suppression in adults with HIV initiating antiretroviral therapy in Tanzania: a three‐arm randomised controlled trial. Lancet HIV. 2020;7:e762–e771. 10.1016/S2352-3018(20)30230-7 32891234 PMC7606811

[29] Linnemayr S , Stecher C , Mukasa B . Behavioral economic incentives to improve adherence to antiretroviral medication. AIDS Lond Engl. 2017;31:719–726. 10.1097/QAD.0000000000001387 PMC5502075 28225450

[30] Chow S‐C , Shao J , Wang H , Lokhnygina Y . Sample size calculations in clinical research. 3rd ed. New York: Chapman and Hall/CRC; 2017. 10.1201/9781315183084

[31] Liu JX , Shen J , Wilson N , Janumpalli S , Stadler P , Padian N . Conditional cash transfers to prevent mother‐to‐child transmission in low facility‐delivery settings: evidence from a randomised controlled trial in Nigeria. BMC Pregnancy Childbirth. 2019;19:32. 10.1186/s12884-019-2172-3 30651080 PMC6335681

[32] Nery JS , Rodrigues LC , Rasella D , Aquino R , Barreira D , Torrens AW , et al. Effect of Brazil's conditional cash transfer programme on tuberculosis incidence. Int J Tuberc Lung Dis. 2017;21:790–796. 10.5588/ijtld.16.0599 28633704 PMC6082337

[33] Ohrnberger J , Fichera E , Sutton M , Anselmi L . The worse the better? Quantile treatment effects of a conditional cash transfer programme on mental health. Health Policy Plan. 2020;35:1137–1149. 10.1093/heapol/czaa079 32879960 PMC7810405

[34] Pega F , Liu SY , Walter S , Pabayo R , Saith R , Lhachimi SK . Unconditional cash transfers for reducing poverty and vulnerabilities: effect on use of health services and health outcomes in low‐ and middle‐income countries. Cochrane Database Syst Rev. 2017;2020:CD011135. 10.1002/14651858.CD011135.pub2 PMC6486161 29139110

