# Supplementary material for: E = mc 2: Education (E), medication (m), and conditional cash (c 2) to improve uptake of antiseizure medications in a low‐resource population: Protocol for randomized trial
Source: Epilepsia Open. 2024 Jan 4;9(1):445–54. doi: 10.1002/epi4.12889 (PMC10839367; doi:10.1002/epi4.12889)
Supplement: Supplementary file 1 — Appendix S1. [file EPI4-9-445-s002.pdf]

Screening Survey (E = mc^2 Study)

Directions: Survey will be administered by trained investigators. As needed, questions will be read to the patient in the patient's preferred language.

Name of potential participant: ____

Investigator name and signature: _____________________________________

Date: ______________ (DD/MM/YYYY)

Time: ______________

Informed Consent and Eligibility Criteria

In order to be eligible for this study, certain criteria must be met:

Is the person a resident of the Republic of Guinea?	*Yes*	No
Is the person 18 years old or older?	*Yes*	No
Is the person able to provide his/her informed consent or, if not, have a next of kin proxy able to provide informed consent?	*Yes*	No
Is the person able to provide a clinical history or have a next of kin provide it?	*Yes*	No
Is the person willing to have their data sent anonymously to the coordinating U.S. study site?	*Yes*	No
Is the person able to undergo the study procedures?	*Yes*	No
Has the person experienced 2 or more recurrent, unprovoked seizures in the past year (365 days)?	*Yes*	No
Is the person currently pregnant or lactating?	Yes	*No*
Is the person critically ill?	Yes	*No*

Is the person taking a daily anti-seizure medicine for the past 30 days or more for any reason?	Yes	*No*
Is the person able to return for follow up visits for 360 days?	*Yes*	No
Does the person have any other diagnosis that can better explain the symptoms?	Yes	*No*
Does the person have seizures due to a serious medical condition other than epilepsy (e.g. malignancy, alcohol withdrawal)?	Yes	*No*
Is the person willing and able to safely undergo a brain MRI?	*Yes*	No
Does the person have a diagnosis of exclusively pseudo-seizures/non-epileptic behavioral events?	Yes	*No*
Is the person willing to take anti-seizure medicines for epilepsy?	*Yes*	No
Is the person already treated with anti-seizure medicines for other purposes (e.g. mood, pain)?	Yes	*No*
Does the person's brain MRI show a secondary cause of seizures (e.g. tumor)?	Yes	*No*

Italicized answers are consistent with study eligibility.
