# Supplementary material for: E = mc 2: Education (E), medication (m), and conditional cash (c 2) to improve uptake of antiseizure medications in a low‐resource population: Protocol for randomized trial
Source: Epilepsia Open. 2024 Jan 4;9(1):445–54. doi: 10.1002/epi4.12889 (PMC10839367; doi:10.1002/epi4.12889)
Supplement: Supplementary file 2 — Appendix S2. [file EPI4-9-445-s001.pdf]

Enrollment Survey (E = mc^2 Study)

Directions: Survey will be administered by trained investigators. As needed, questions will be read to the patient in the patient's preferred language.

Demographic information

Name of patient: ______________________________ **Patient #:** ____________

Date of evaluation: _____/_____/_____ (DD/MM/YYYY) **Name of evaluator:** ________________

Current residence (village): ____________________ **District:** ______________________

Hours traveled to clinic: ____________________

Primary phone number: ____________________________

Phone number details (personal, family member, friend, etc.): ________________

Secondary phone number: ____________________________

Email: ____________________________

Preferred method of contact: ____________________________

Age (years): _______ **Date of birth:** _____/_____/_____ (day/month/year)

Gender (circle one): Male Female Nonbinary

Highest education level completed (circle one):
No school Primary school Secondary school High school University

Currently in school: Yes / No

Religion (circle all that apply):
Muslim Christian Other: ______________________

Occupation ("none" or list): ________________________________

Medical History

How old were you when you had your first seizure (years): ________

Have you been diagnosed with epilepsy? Yes No

- **If yes, how old were you when diagnosed (years):** ________

- **If yes, who diagnosed you? (circle all that apply)**

medical doctor (western/modern) traditional healer other: ____________________

How would you characterize your seizures? (Check all that apply)

□ loss of consciousness
□ falling to ground with stiffening and shaking of body
□ falling to ground, no shaking
□ uncontrollable shaking of one part of the body
□ staring spells
□ unusual behavior or acting strangely
□ communicating with spirits
□ unusual sensory events (vision hearing touch smell)
□ tongue biting
□ urinary incontinence
□ salivation
□ grunts/shouting/noises of some form
□ other: ____

How many seizures have you had in total?: _______________________________________

How many seizures have you had in the past month?: _______________________________________

When was your last seizure? (check one)

□ within the last week □ within the last month
□ within the last 3 months □ within the last 6 months
□ within the last 9 months □ within the last year
□ over 1 year ago

Do any of the following trigger your seizures? (check all that apply)

□ infections/fever □ lack of sleep □ flashing lights □ stress □ alcohol
□ forgetting to take medications □ other: _______________________________________

Seizure Treatment

Have you ever taken anti-seizure medicines for epilepsy? Yes No

If yes, why did you stop taking the medication: _______________________________

When did you first start anti-seizure medicines for seizures? (Age, in years): ________

Which medications or treatments? (check all that apply)

□ Phenobarbital Dose: ____________________
□ Phenytoin Dose: ____________________
□ Carbamazepine Dose: ____________________
□ Sodium Valproate Dose: ____________________
□ Levetiracetam Dose: ____________________

□ Clonazepam Dose: ______________________

□ Clobazam Dose: ______________________

□ Diazepam Dose: ______________________

□ Lamotrigine Dose: ______________________

□ Gabapentin Dose: ______________________

□ Other: ____

List any side effects from medications:

____

Have you ever attempted other treatments for seizures?

□ Special diet	Explain: ______________________	□ Currently taking	□ Not anymore
□ Traditional treatments	Explain: ______________________	□ Currently taking	□ Not anymore
□ Prayer		□ Currently taking	□ Not anymore
□ Other	Explain: ______________________	□ Currently taking	□ Not anymore

Have you ever had:

□ Head CT Date: ________________

Results: ____

□ Brain MRI Date: ________________

Results: ____

□ EEG Date: ________________

Results: ____

Family & Past Medical History

**Does anyone else in the family (blood relatives) have seizures?**	Yes	No
If yes, does *more than one* family member have seizures?	Yes	No
**Have you ever had a head injury with loss of consciousness?**	Yes	No
**Have you ever had a stroke?**	Yes	No
**Have you ever had meningitis?**	Yes	No
**Have you ever had cerebral malaria?**	Yes	No

Do you have neurocysticercosis*?* Yes No

Do you drink alcohol? Yes No

If yes, do you drink more than 2 alcoholic beverages a day? Yes No

If yes, do you drink more than 14 alcoholic beverages a week? Yes No

If yes, have you ever had a seizure after stopping drinking alcohol? Yes No

What other medical problems do you have? ____

____

Are you on any other medications or treatments? Yes / No

If yes, medication name: ______________________________

Indication: ______________________________

Date started: ______________________________

Have you ever had any injuries related to seizures? (check all that apply)

- ◻ Burns
- ◻ Breaking bones/fractures or bone dislocation
- ◻ Head injury
- ◻ Car accidents
- ◻ Skin injury (scratches, cuts)
- ◻ Other: ____

Do you drive a car/motorcycle/truck? Yes No

For women: have you ever had a seizure during pregnancy? Yes No

For women: did you take anti-seizure medicines while you were pregnant? Yes No

If yes, which medicines? ____

<u>Consumption and household assets</u>

The following questions are designed for us to understand more about your financial situation, as this can affect the medical care that individuals are able to access. When asked for a monetary value please answer in Guinean Francs.

Is there a head of the household? If so, what is their relation to you? ______________________________

What is the highest education of the head of the household:

- ☐ No school
- ☐ Primary school
- ☐ Lower secondary

- ☐ Upper secondary
- ☐ University
- ☐ Unknown

How many people are there in your household: ________

Number of adults 18 and over: ______

Number of adults who are working: _______

Number of adults who are not working: _______

Number of children: _______

Number of older (non-working) adults: _______

How much money have members of your household earned in the past month? ________

In a typical month, how much money does your household spend to cover all expenses including housing, food, schooling, and household goods? _________

In a typical month, how much does your household spend on food? ________

In a typical month, how much does your household spend on schooling? ________

In a typical month, how much does your household spend on treatment for epilepsy, including medications, visits to doctors, and traditional therapies? _________

Cost of Anti-epileptics ________
Cost of visits to doctors _________
Cost of traditional therapies ________

What is the marital status of the head of the household?

- ☐ Married or living together
- ☐ Divorced or separated
- ☐ Widowed
- ☐ Never married

Is your mother currently alive?

- ☐ Yes
- ☐ No

Is your father still alive?

- ☐ Yes
- ☐ No

Where is the water supply?

- ☐ In own dwelling
- ☐ In own yard/plot
- ☐ Elsewhere

How long does it take you to go there, get water, and come back?
Minutes: ________
[ ] Don't know

What is the main source of drinking water for members of your household?

- ☐ Piped water (tap water)
  - o Piped into dwelling
  - o Piped into yard/plot
  - o Piped into neighbor
  - o Public tap/standpipe
- ☐ Tube well or borehole
- ☐ Dug well
  - o Protected well
  - o Unprotected well
- ☐ Water from spring
  - o Protected spring
  - o Unprotected spring
- ☐ Rainwater
- ☐ Tanker truck
- ☐ Cart with small tank
- ☐ Surface water (rivers/dams/lakes/ponds/rivers/canals/irrigation channel)
- ☐ Bottled water
- ☐ Other: __________

What is the main source of water used by your household for other purposes such as cooking and handwashing?

- ☐ Piped water (tap water)
  - o Piped into dwelling
  - o Piped into yard/plot
  - o Piped into neighbor
  - o Public tap/standpipe
- ☐ Tube well or borehole
- ☐ Dug well
  - o Protected well
  - o Unprotected well
- ☐ Water from spring
  - o Protected spring
  - o Unprotected spring
- ☐ Rainwater
- ☐ Tanker truck
- ☐ Cart with small tank
- ☐ Surface water (rivers/dams/lakes/ponds/rivers/canals/irrigation channel)
- ☐ Bottled water
- ☐ Other: __________

In the past two weeks, was the water from this source not available for at least one full day?

- ☐ Yes

☐ No
☐ I don't know

Do you do anything to the water to make it safer to drink?

☐ Yes
☐ No
☐ I don't know

What do you usually do to make the water safer to drink? Please check all that apply.

☐ Boil
☐ Add Bleach/Chlorine
☐ Strain through a cloth
☐ Use water filter (ceramic/sand/composite/etc)
☐ Solar disinfection
☐ Let it stand and settle
☐ Other _______________
☐ Don't know

What kinds of toilets to members of your household typically use?

- ☐ Flush or pour flush
  - Connected to piped sewer system
  - Connected to a septic tank
  - Connected to a pit latrine
  - Connected to something else
  - I don't know what it's connected to
- ☐ Latrine
  - Ventilated improved pit latrine
  - Pit latrine with slab
  - Pit latrine without slab/open pit
- ☐ Composting toilet
- ☐ Buckets
- ☐ Hanging toilet/ Hanging latrine
- ☐ No toilets/bush/field
- ☐ Other: __________________

Do you share this toilet with other households?

- ☐ Yes
- ☐ No

Including your own household, how many households use this toilet facility?

☐ Number of households __________
☐ More than 10
☐ Not sure

Where is this toilet facility located?

☐ In own dwelling
☐ In own yard/plot
☐ Elsewhere

Does anyone in your household have a bank account?

- ☐ Yes
- ☐ No

Please check the box if you do have the following items in your household:

- ☐ Electricity
- ☐ Radio
- ☐ Television
- ☐ Non-mobile telephone
- ☐ Computer

Please check the box if any member of your household owns the following:

- ☐ Watch
- ☐ Mobile phone
- ☐ Bicycle
- ☐ Motorcycle or motor scooter
- ☐ Animal drawn cart
- ☐ Car or truck
- ☐ Boat with a motor

Does your household have any mosquito nets?

- ☐ Yes
- ☐ No

<u>Clinical measurements</u>

Body weight: _________

Height: _________

Heart rate: _________

Serum Na+ (as needed): _________

Serum glucose (as needed): _________

Pregnancy test results (for women of childbearing potential): _________

MRI imaging results (append to survey)

If you have any comments, please write them here: ____

____

____

____

____

____
